# Patient and Public Perceptions of 3D Technologies (Models and Images) to Facilitate Health Care Consultations: Exploratory, Mixed Methods Study

**DOI:** 10.2196/65235

**Published:** 2025-06-18

**Authors:** Harleen Kaur Rai, Morven Miller, Steve Leung, Euan Macleod, Marilyn Lennon

**Affiliations:** 1 Department of Computer and Information Sciences, University of Strathclyde, 16 Richmond Street, Glasgow, G1 1XQ, United Kingdom, 44 0141 548 4303; 2 Urology Department, Western General Hospital, Edinburgh, United Kingdom; 3 Directorate for Strategic Planning and Transformation, Dumfries and Galloway NHS, Dumfries, United Kingdom

**Keywords:** 3D technology, citizen science, public and patient involvement, health care, public perceptions, health care consultations, consultations, mixed methods study, information, shared decision-making, acceptability, innovative technology, web-based survey, public health, telephone interviews, willingness, qualitative study

## Abstract

**Background:**

3D technology, including models and images, can facilitate health care consultations by promoting a better understanding of information by patients and shared decision-making. However, little is yet known about the general public’s perspectives about the acceptability of such innovative technology and how it can best be adopted into routine health care consultations. There is a need to explore both public and patient perceptions to avoid the risk of implementing 3D technologies that may not be acceptable or fit-for-purpose.

**Objective:**

This paper aimed to explore the patient and public perceptions of the use of 3D technology during health care consultations.

**Methods:**

This study adopted a citizen science approach using mixed methods to conduct (1) a short web-based survey with members of the public to gather a wide range of opinions regarding the use of various technologies for health care consultations; (2) a longer web-based survey to explore perceived barriers and opportunities people report specifically on the use of 3D technology; and (3) telephone interviews with patients who recently used 3D technology as part of their health care consultations.

**Results:**

A total of 211 participants completed the short survey, of which 25 went on to complete the longer survey. While members of the public were familiar with using various types of technologies during remote consultations, most participants did not have experience with using 3D technology. However, people reported that they could see the potential benefits of such technology to facilitate health care consultations. They expressed positive perceptions toward how this might assist in comprehension of a diagnosis and discussion of alternative treatment plans. They also mentioned potential benefits in relation to communication and shared decision-making either with their health care provider or with their friends and family. These potential benefits were confirmed through telephone interviews with 4 patients who also stressed potential barriers such as emotional distress caused by an overload of information as important considerations for wider implementation. Overall, there was a strong interest and willingness to use 3D technology in future health care consultations.

**Conclusions:**

The use of 3D technology in health care settings is now an option, but there is little research to date on how patients and the wider public might benefit from this. This mixed methods study has shown that people are accepting of 3D technology being used in health care consultations and that there might be real benefits to the patient. These include improved individual and shared decision-making around their treatment through the technology, making disease and treatment options easier to understand for patients. Since 3D technology can still be expensive, the benefits to the patient and health care professionals need to be captured and quantified in terms of reduced travel, efficient use of time, and overall better quality of care and clinical outcomes.

## Introduction

**Figure 1.**
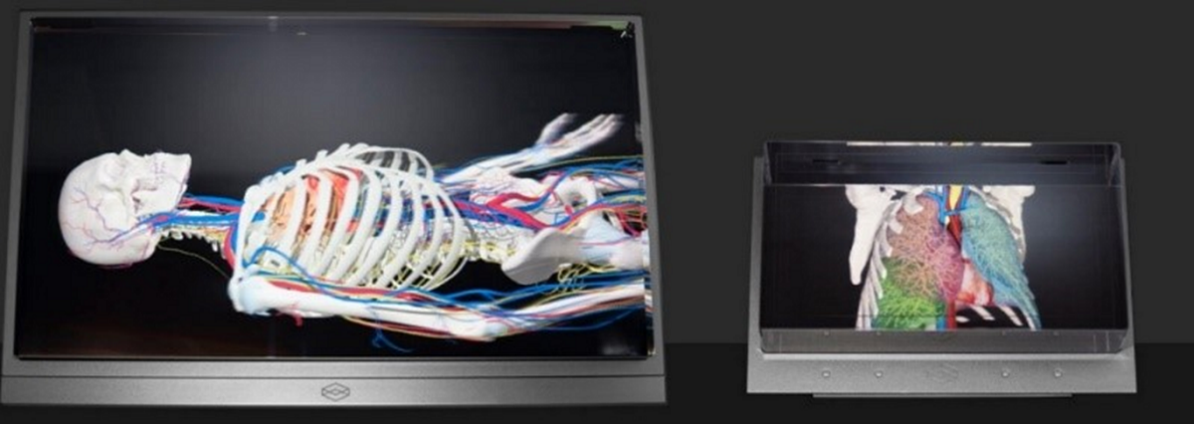
Example of a 3D health care model showcasing detailed views of the skeletal and organ system [9]. Used with permission from Holoxica.

Patients are often given information in many forms to encourage them to be involved in the decision-making process about their own health. Particularly in surgical disciplines, diagnostic imaging information such as computerized tomography, magnetic resonance imaging, or radiograph (x-ray) scans can be used to illustrate the patient’s anatomy and condition [1]. These types of images or information can be hard for most patients to fully understand, especially in cases of low levels of health literacy [2]. The use of more advanced digital technologies might be helpful for sharing this complex information with patients and aiding their overall understanding of their condition and treatment options [3]. This implementation of digital technologies also fits within the recent shift toward remote or virtual consultations in light of the COVID-19 pandemic, a change that arguably has a significant impact on doctor-patient interactions [4]. Many specialties undertook new strategies during this time, such as making further use of telephone clinics and videoconferencing platforms such as Teams or NHS Near Me to conduct clinical meetings [4,5]. There is a clear opportunity to use new technologies, but only if they are acceptable to patients and provide advantages above the “standard” face-to-face consultation in the clinical setting. Clinicians often use 2D images to aid patient discussions of their disease. With the expanding roll-out of 5G telecommunications; however, 3D technologies such as images, models (physical replicas of the disease), and holographic displays are now emerging and, if successful, could significantly improve the consultation experience for patients, carers, and clinicians by reducing costs, travel and waiting times and contributing to the green agenda [6,7]. The latter would allow for expert care to be delivered closer to the patient’s home in line with the Scottish Government’s delivery plan for Care in the Digital Age [8]. 3D technologies can often be used without a headset or specialized virtual reality eyewear meaning that the patient and clinician are not inhibited by cumbersome virtual reality equipment offering a far less physically and socially restricting environment to view 3D visualizations during a consultation. 3D technologies can convey information of the disease to the patient in novel ways, for example, a 3D image viewed on a monitor can create a depth of field making a complex object more understandable (see Figure 1).

By presenting the information in a novel way, clinicians can effectively explain complex procedures, highlight risk and support the discussion of treatment options potentially leading to a level of joint understanding of the disease state and helping the patient make choices about their future management [2]. In the field of renal cancer surgery for example, Wake et al [2] demonstrated that patient understanding, and education improves with the addition of 3D models in a consultation. Several 3D imaging methods were used but 3D printed models were noted to have the greatest impact on patient understanding of their disease, tumor characteristics and the surgery itself. The ability to see the tumor in 3D can also make the patient feel more involved in their treatment and leads to greater trust in the clinician and their surrounding team [10]. Consequently, 3D models can play a great part in strengthening the shared decision-making process that is rapidly becoming the cornerstone of modern medicine [11,12]. For clinicians, the use of 3D technology provides an additional benefit: it may improve surgical outcomes by allowing surgeons to plan and rehearse their surgery on personalized models of their patients’ pathology. This may have the benefits of shorter operative time, more accurate surgery leading to higher rate of curative surgery, and reduced blood loss leading to quicker recovery [13].

With advancing technologies come huge opportunities to enhance the way the public interacts with professionals and health and care systems. However, while there is some research demonstrating the benefits of 3D technologies from a patient perspective, there is not much known about the public’s readiness to adopt such technologies as part of standard care. For 3D technologies to be fit-for-purpose and implemented more widely, there is a need to understand what the general public’s perceptions and expectations are from these technologies. Without this, there is a risk of adopting technologies which will exclude people rather than improve their interactions and their health outcomes. Therefore, the aim of this study was to explore not just patient but also wider public perceptions of the use of 3D technologies including images and models during health care consultations.

## Methods

### Design

This was a mixed methods study using surveys and telephone interviews. We adopted a citizen science approach where we set out to actively include voices and experiences of the public as well as people who have had experience of 3D technology in their recent health care interactions. Giving people a chance to provide their lived experiences is important for increased buy in, adoption and scaling of technologies in the future. A real-world service evaluation approach guided this study to gather a range of opinions on an emerging technology and how it might be adopted and received by people in their day to day lives. The aim was not to evaluate or validate the technology itself but to explore whether technology such as this was acceptable and so could be adopted into routine care consultations—specifically from the patients’ perspective of potential benefits and barriers. Such evaluations of acceptance prior to adoption and scale are essential to test for change to maximize the chances of the technology working in practice and to avoid human factors issues that might arise if effective technologies are simply rolled out without prior consultation with the public and patients.

### Participants

Different user groups were recruited for the various stages of this study. Participants from the public aged 18 years or older were invited to complete a short web-based survey. Participants who had completed the short survey were then asked to share their email address if they were interested in completing a longer survey to elaborate on their previous responses. Telephone interviews were conducted with participants who were aged 18 years or older, who had lived experience of receiving a diagnosis and treatment for kidney cancer and had experience with using 3D technology as part of their care consultations.

### Ethical Considerations

Ethical approval was obtained from the University of Strathclyde's Department of Computer and Information Science Ethical Committee (ref: 1797). All participants were provided with an information sheet, included as part of the web-based surveys or via email if participating in telephone interviews. Written or verbal informed consent was obtained from all participants prior to taking part in the surveys or the telephone interviews. Survey participants had the option to share their email addresses, for opting into the long survey. No other personally identifiable data were collected. Data from the telephone interviews were deidentified during analysis. Participants were not compensated for their involvement, but a £50 (US $67.94) gift voucher was awarded to 5 people who completed the short survey and 10 people for the long survey using a digital random number generator.

### Recruitment

A convenience sampling approach was used to recruit participants from the public. Invitations to participate in the short survey (see Multimedia Appendix 1) were advertised via social media including personal Twitter accounts of members of the research team as well as sent directly to various charities and carer groups (including Action Kidney Cancer, Fibromyalgia Action UK, Myeloma UK, MacMillan Cancer support, and Carers Scotland). Recruitment for the long survey was facilitated by participants opting in after completing the short survey. Both surveys were completed in the web-based survey tool named Qualtrics. Recruitment for the telephone interviews took place in a primary care setting. A Consultant Urological Surgeon, who was part of the research team, assessed recent patients against the study’s eligibility criteria and invited eligible patients from his clinics with lived experiences of a kidney cancer diagnosis to participate in a short telephone interview to evaluate the service. Patients were asked to describe their experiences with 3D technology (eg, images or models) which had been used during their consultation.

### Data Collection Short Survey (Survey 1)

A survey including 4 short multiple choice and 4 open-ended questions (see Multimedia Appendix 2) was advertised digitally and shared with third sector organizations to gather a wider range of opinions regarding the use of various technologies (including 3D technology) for health care consultations. The aim was to gather opinions from a more diverse range of people with a range of needs, preferences, and demography. The survey was available for completion for 5 months (March to August 2022).

### Long Survey (Survey 2)

A longer survey including a mix of 17 multiple choice and open-ended questions was distributed to people opting in from the short survey. Participants were invited to elaborate on previous responses and to share their stories with us in terms of their experiences of and attitudes toward new 3D technology for health care consultations. The survey was available for completion for one month (September to October 2022).

### Case Studies With People With Lived Experience

Four patients with lived experience of kidney cancer surgery participated in individual, telephone-based interviews to explore their actual experiences of using 3D technology such as images or models during consultations for diagnosis or treatment. The research team developed an interview topic guide (see Multimedia Appendix 3) including 5 open-ended questions related to general experience of using 3D technology, benefits, barriers, and future use for such technology. Questions were followed up with probes where appropriate (eg, specific impacts). Each interview was audio-recorded and lasted between 10 and 20 minutes.

### Data Analysis

Numerical data from the surveys was presented descriptively using the inbuilt descriptive summary reporting tools in Qualtrics. This included the frequency of responses in each category. Free text responses were examined by 2 members of the researcher team (HKR, MM) to thematically categorize respondents’ perceptions. The free text analysis was mostly deductive in nature as our a priori themes were pre-defined by the questions in our survey which were centered around our research aims. Any emerging themes that were additional to our a priori themes were included as additional perceptions. Data from the telephone interviews were not transcribed, rather the research team (HKR, ML, MM) made extensive notes of the audio-recordings and these were summarized thematically by the lead researcher (HKR).

## Results

### Quantitative Results From Survey 1 & 2

A total of 253 participants took part in the first, short survey in part of which 211 participants completed it in full. Most participants had experience with taking part in a remote consultation with a health care provider. This was mostly done through phone calls (n=123) or video calls (n=62) (see Table 1).

**Table 1.** Results from the short survey showing an overview of remote consultation methods used by members of the public during health care consultations.

Answer	Count, n (%)
Yes—phone call (eg, a landline or your mobile phone)	123 (48.62)
Yes—video call (eg, this includes Skype, Microsoft Teams, Facetime, WhatsApp, Zoom)	62 (24.51)
Yes—email (eg, discussing your care with a health professional)	51 (20.16)
Other—please describe below	1 (0.40)
No	16 (6.32)

Both modalities were rated as “quite good” or “extremely good” by most participants in terms of their usability, performance, and helpfulness but overall, video calls were rated more positively than phone calls on all aspects. In relation to different types of 3D technology, 62 participants had heard of it and were interested in its use.

A total of 25 participants from the short survey went on the completed the long survey in full. None of the participants had any direct experience with using 3D technology in their interactions with a health care consultant. Despite this lack of experience, the majority agreed such technology could potentially be useful (n=17), improve understanding of one’s condition (n=13) and treatment options (n=10), help to understand surgical procedures (n=14) and their associated risks (n=14), impact personal decision-making for treatment (n=11), help with sharing information with family and friends (n=13), and impact the relationship with their health care provider (n=13) .

### Qualitative Results From Survey 1 & 2

The free-text results of both surveys were reviewed and are presented below according to the following themes: understanding and sharing information; relationships; barriers and concerns; and benefits and opportunities (current and future).

#### Understanding and Sharing Information

Within survey 1, respondents acknowledged the usefulness and helpfulness of all technologies (telephone, video call, email, Near Me app) for health and social care consultations. Being able to see the health professional in a video call was felt to promote greater understanding than a simple telephone call. The helpfulness of emails to keep a record of health details and prepare questions to ask of the health professional was noted. Respondents identified specific occasions when technology would be appropriate for sharing information. For example, for routine, non-urgent appointments such as medical reviews, discussing blood results, and when someone is unable to travel. It may also be useful in facilitating conversations to determine whether in person consultations are needed for discussing a change in health status, more serious conditions, sensitive or detailed decisions or mental health issues.

Those who responded to the second survey reported that 3D models would be helpful to increase their understanding of their condition and treatment options primarily because of the increase in clarity and detail of information available. 3D models were seen to make information about a diagnosis more real and relatable. The potential of visualization was also noted in relation to understanding the impact of treatment, for example, comparing between treatments or whether a treatment had been effective. The deeper understanding from 3D models would help people to ask questions about their diagnosis and treatment options, reducing their need to understand medical jargon. Respondents noted that the information from 3D models could help mitigate the potential for misunderstandings as well as being helpful for sharing with family members and friends as, if they understood their condition better, they would be better able to communicate this to others.

Beyond the sharing of information between patients and health professionals within a consultation, the use of 3D models was also seen as a more general educational opportunity for health professionals, patients and for use in educating people in schools.

#### Relationships

Within survey 1, respondents identified a lack of face-to-face connectedness as a negative outcome of using technology for consultations, in particular losing the subtleties of visual cues and the inability to conduct physical examinations. While emailing a photograph was seen as a workaround by some, it was not thought to be acceptable to others. Within survey 2, the use of 3D models was consistently seen to improve the potential relationship that a patient would have with their health professional by creating a sense of partnership between the two as well as increasing a patient’s confidence and trust in their health professional. The concept of 3D models helping to overcome the use of medical jargon was raised again in relation to improving relationships between patients and health professionals and this reduced use of jargon was seen to make it easier for patients to ask questions of their health care professional.

#### Barriers and Concerns

Within survey 1, respondents identified a lack of access to the appropriate equipment and facilities (such as connectivity), as well as how to use them, as a barrier to remote health and social care consultations. Wasted time waiting for a phone or video call was noted as a downside of remote consultations as was waiting for a response to an email.

Within survey 2, some respondents noted that the increased information provided by 3D models may be scary or provide too much information for some people and so increase their sense of anxiety. Visual or cognitive impairment, apathy to technology and advanced age were all identified as factors that may act as barriers to the use of 3D models in practice. Resource and practical issues including associated costs, availability, time taken to produce models and complexity of IT required were also noted to be issues that may constrain the use of 3D models. It was also noted that 3D models would not be helpful for facilitating discussions about all diagnoses (eg, hematological cancers) or treatment options. Another challenge identified was the capability of the technology to be used for remote consultations. The fact that not everyone wants to share this type of information with family members or friends was also highlighted. The environmental impact of producing 3D plastic models was also mentioned. Finally, that care and compassion should not be lost in interactions between patients and health professionals using such technology was an important consideration.

#### Benefits and Opportunities (Current and Future)

Respondents to survey 1 noted many benefits of technology-facilitated consultations relating to time: saving time in general, being at a convenient time; avoiding the inconveniences and time associated with travelling to appointments; responding to emails at a time that suited them. Remote consultations were seen to be a more convenient and cheaper option than travelling to an appointment. There was an overwhelming sense from respondents to survey 2 that 3D models offered benefits across multiple health care scenarios, for example: diagnosis; presurgical and decision-making consultations. Overall, implementing this technology where possible and appropriate was seen as a positive addition to interactions between patients and health professionals.

### Case Studies (Lived Experience)

#### General Experience

Most participants had experience with both a 3D image and 3D model during successive care consultations. For instance, one participant was shown a 3D image as part of a general consultation and a 3D model was presented at a later stage to provide more details regarding the upcoming surgical procedure. Some of these consultations had to take place remotely due to the COVID-19 pandemic meaning participants were able to see 3D images at home and 3D models were sent by post where needed. An added benefit of these remote consultations included the involvement of family members as they were able to join a Near Me call or could handle the 3D model at home which was perceived to promote shared understanding. However, 2 participants mentioned they preferred seeing a 3D model in a care setting only as they wanted to avoid revisiting issues surrounding their health at their home.

All participants were positive about their experience of using 3D images and 3D models. Most notably, participants felt that such 3D technology provided a better perspective on their diagnosis and as a result felt reassured, less anxious, and more confident fuelled by a better understanding, eg, regarding the size and location of the tumor or treatment procedure:

*I made my mind up when I’d seen it, that I was not going to die. […] It was very reassuring.*

[Interview 1]

Three participants mentioned that conventional scans are not very clear or understandable and that a 3D image on the screen or holding a 3D model were both much clearer:

*The 3D image that he showed me initially was ten times better than the scan […] it brought everything to life.*

[Interview 2]

One participant also mentioned that ‘to actually see the image’ or ‘to feel the model’ was better than reading or listening about her condition as she is dyslexic.

#### Benefits

All participants shared several benefits of which improved understanding of diagnosis and treatment was mentioned most often.

*I think it takes a lot of the fear away.*

[Interview 1]

The technology helped to pull together all the information patients had already received by enabling visualisation of treatment procedures and consequences through images rather than having to understand and remember information that was given verbally or on paper only.

Could we say “by enabling me to visualise the procedure and see it presented as images rather than having to remember and try to understand what was said to me.”

This increased understanding also had a positive impact on the relationship with their clinicians, for example, increased trust and confidence. Two participants mentioned they felt that they were receiving more personalized care and were seen as individuals rather than just another patient.

*They are doing all they can to benefit me.*

[Interview 3]

Other benefits included improved information sharing and communication. This was particularly helpful when sharing details surrounding a diagnosis with family members and friends who often themselves were concerned for the participant’s well-being. For one participant their own improved understanding helped to understand what was happening to a family member who was also receiving treatment for kidney cancer. Lastly, when it came to decision-making, for most of the participants, this was not relevant as a decision had been made prior to seeing 3D images and models. However, one participant mentioned that he felt less anxious when making decisions though he could not say for sure whether this was due to a good relationship with his clinician or the 3D technology.

#### Barriers

While participants did not experience any challenges or difficulties with the 3D images or models, some recognized that some people may feel upset or distressed from being presented with such 3D models. For instance, one participant thought that detailed 3D technology may induce anxiety in some patients due to an overload of detailed information and that improving people’s understanding of their diagnosis and/or treatment could lead to increased anxiety as it would ‘make things very real’. However, for the same participant this barrier was seen as a benefit as they preferred to be in possession of all the available information:

*It could give an information overload […] it makes things very real for them but for me this was an absolute benefit.*

[Interview 3]

#### Future Use

When asked about when 3D images or models would be most useful, one participant mentioned they would be helpful at any point during the cancer journey. Others mentioned 3D technology could be most useful when discussing options available for treatment and/or surgery. Some participants felt that it would not be useful at the point of receiving a cancer diagnosis or during a first consultation as patients may need to come to terms with receiving their diagnosis before being presented with additional information through 3D technology as this could be overwhelming.

However, all participants agreed that 3D images and models held a lot of potential and that they should be implemented more widely within health care settings in the future beyond cancer consultations. Some suggestions for future clinical use included showing broken bones, brain injuries, or other types of surgery (not just urology). One participant felt that most patients would like to have a 3D image or model presented to them if it was available and reiterated their usefulness:

*It’s a very positive option to have. I would recommend anybody to do it if they have the option.*

[Interview 3]

## Discussion

This study set out to explore patient and public perceptions of the use of 3D technologies during health care consultations to further shed light on its potential for adoption as part of standard care. To the best of our knowledge, this is the first work of its kind in which we integrated a citizen science approach with mixed methodology consisting of surveys and case studies. Overall, our findings show a strong interest and willingness to use 3D technology among members of the public which is further supported by positive patient experiences indicating an encouraging potential for 3D technologies to be adopted as part of standard care.

Our results indicate that members of the public were experienced in the use of various technologies during remote consultations. Most people did so through telephone, video calls and emails and found the added convenience of saving time and travel to be the most important benefit. Also, different types of technologies were also associated with different benefits. For instance, video calls were helpful for a greater understanding of information during routine and non-urgent appointments while emails were thought to be useful for keeping a record of health details. Previous research states that the effectiveness of remote consultations and different types of technologies indeed depends on their purpose. For instance, Greenhalgh et al [14] found that remote consultations were perceived to be more appropriate when engaging in more conventional and routine appointments as opposed to appointments for newer and more urgent cases. However, our findings have also highlighted individual barriers including a lack of face-to-face connectedness, appropriate equipment, and facilities as well as a lack of knowledge or skills on how to use these which is backed by previous research [15]. Individual barriers related to health and access may impact the experience of using technologies during remote consultations. In their narrative synthesis, Walthall et al [16] found that internet-based, remote consultations in general practice are more likely to be used by younger, affluent, and educated groups. Researchers stress the importance of considering (health) inequalities in the wider use and implementation of remote consultations and relevant technologies.

The majority of our study participants were also familiar with the existence of 3D technology such as images and displays as part of health care interactions but had no experience of using such technology in practice. However, participants recognised that such technology could have some wide-ranging benefits leading to a positive impact on the overall health care consultation experience. People mentioned an improved understanding and sharing of information regarding their condition and treatment options as one of the main benefits. 3D technology was considered to increase clarity and the level of detail, making the available information more real and relatable. In addition, people also felt that such technology could enhance the relationship with their health care professional through creating a sense of partnership which could lead to increased trust and confidence in the health care professional. These benefits were also confirmed by patients with lived experience. Patients expressed how 3D technology might assist in comprehension of a diagnosis and in discussing alternative treatment plans. They also mentioned potential benefits in communication and shared decision-making either with their health care professional or with their friends and family. It is important to note that there is a large body of literature surrounding patient experiences of remote health care consultations and of more conventional technologies, especially considering the COVID-19 pandemic, but research on the use of 3D technologies in such consultations with patients is still relatively limited. Studies investigating the use of 3D technology from a patient perspective are particularly lacking. A recent review on the application of 3D technology within health care education highlighted a range of benefits related to optimisation of costs of complex surgical processes as well as reduced time and fewer complexities during operations due to pre-surgical planning using 3D technologies [17]. Similar to our findings, 3D technologies can help to improve the relationship between the patient and health care professional during health care consultations and address any gaps in communication leading to better patient understanding [17]. These benefits are confirmed in an earlier review by Senbekov et al [18] who state that 3D technologies can support patient education and patient-oriented care.

Despite these encouraging benefits, people also felt that for 3D technologies to be implemented widely during health care consultations, some barriers would need to be taken into consideration. For instance, both members of the public and patients with lived experience noted that the increased information provided by 3D technology may lead to an overload of information for some people and so increase their sense of anxiety. Indeed, in an exploratory study with patients with glioma, van de Belt et al [12] found that patients were positive about 3D technologies in terms of improved patient understanding but were also concerned that such technologies could cause emotional distress especially early on in their treatment which was also noted in our research. Members of the public in this study also identified individual barriers such as cognitive impairment and apathy to technology as well as practical and resource barriers including any associated costs and time needed to produce 3D models and their environmental impact that may hinder the wider implementation of 3D technology. As with the use of any technology during remote consultations, people also felt that care and compassion should not be lost in interactions between patients and health care professionals using 3D technology.

This study has several limitations. Given that the surveys were disseminated through selected charities and carers groups, this may have caused selection bias during recruitment leading to our sample lacking full representation of the wider public. Furthermore, we recruited using web-based methods and organized a web-based survey which excluded people without access to the internet further impacting the generalizability of our research findings. In addition, positive experiences regarding both remote consultations in general as well as (the potential of) using 3D technology during such consultations were overrepresented in our data and a more diverse approach to sampling may have helped to capture more nuanced and negative experiences and opinions. However, both the sampling process and the relatively small sample size were appropriate for an exploratory study and allowed for convenient data collection that highlighted potential willingness, positive attitudes and valuable insights toward using 3D technologies during health care consultations that others could find helpful in considering whether the findings may be relevant in their settings. Though we did not capture demographic data in our survey and case studies, this is particularly relevant when considering population groups that face health inequalities (eg, people with lack of access to technology, low levels of literacy, ethnic minority groups) have experiences with remote consultations and 3D technology that are likely to differ. Finally, our case studies did not include detailed transcription and thematic analysis which may have limited the amount of rich and in-depth data we were able to capture.

In conclusion, the use of 3D technology in health care settings is now an option but there is little research to date on patient experiences with such technology and how the wider public might benefit from this. There is an overwhelming sense from our research that 3D technology offers wide-ranging benefits with a potential for application across multiple health care scenarios. People are accepting of 3D technology being used in health care consultations and there may be real benefits in practice including improved patient understanding of diagnosis, treatment options, and risks leading to enhanced individual and shared decision-making as well as a better relationship with the health care professional. More research is needed to better quantify these benefits to the patient and health care professionals but also in terms of cost savings through reduced travel, efficient use of time, and overall better health outcomes. Lastly, with the wider implementation of 3D technologies, barriers surrounding accessibility need to be considered to prevent the exclusion of more vulnerable populations from using 3D technology.

## Supplementary material

10.2196/65235 Multimedia Appendix 1 Recruitment flyer for short survey.

10.2196/65235 Multimedia Appendix 2 Short survey.

10.2196/65235 Multimedia Appendix 3 Case study interview guide.
