# Supplementary figures and images for: Patient and Public Perceptions of 3D Technologies (Models and Images) to Facilitate Health Care Consultations: Exploratory, Mixed Methods Study
Source: JMIR Form Res. 2025 Jun 18;9:e65235. doi: 10.2196/65235 (PMC12192911; doi:10.2196/65235)

**Multimedia Appendix 1 - recruitment flyer for short survey**


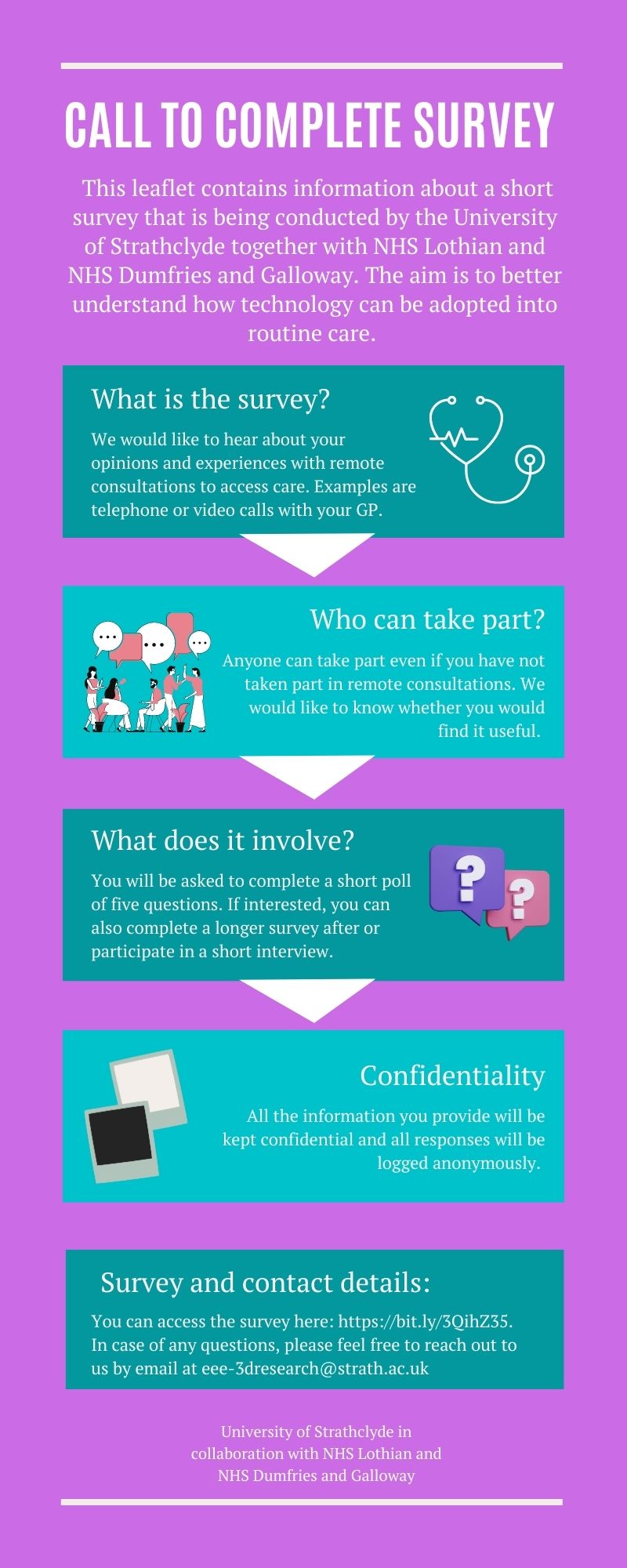

Supplement: Multimedia Appendix 1 [file formative-v9-e65235-s001.docx]
