# Supplementary material for: Patient and Public Perceptions of 3D Technologies (Models and Images) to Facilitate Health Care Consultations: Exploratory, Mixed Methods Study
Source: JMIR Form Res. 2025 Jun 18;9:e65235. doi: 10.2196/65235 (PMC12192911; doi:10.2196/65235)
Supplement: Multimedia Appendix 2 [file formative-v9-e65235-s002.docx]

**Multimedia Appendix 2 - short survey**

Thank you for agreeing to complete this short survey being conducted by the University of Strathclyde in Collaboration with NHS Lothian and NHS Dumfries and Galloway. It is anonymous and should only take 5-10 minutes to complete. The results we receive will be used to help us understand people’s perceptions about the use of 3D displays during consultations with health and care professionals.

Privacy and Consent

By clicking next at the bottom of this page you agree that: You are aged 18 or over. You understand what is being asked of you and you are happy to complete the short survey. You understand that your data will be treated confidentially and not shared with any third parties. You understand that you will not be individually identifiable from your data. You are aware that the email address you provide at the end for being included in the prize draw will be stored separated from your actual data and only used to contact you if you win the prize draw. You are happy that if you opt in to be contacted for our next phase of data collection we will only use your email address to contact you to provide further information for phase 2 where you will be given further time to decide whether or not you want to take part in the next stage. By clicking next you are agreeing to take part in this short survey.

Please complete the following four short questions about the use of technology (e.g. laptops, phones, video calls, computer displays) to support remote communication/consultations in the delivery of your health and care (e.g. consultations, appointments, check-ups).

1. Have you ever taken part in a remote appointment with a healthcare provider (e.g. doctor, nurse, pharmacist) that used technology, such as a telephone or video call (either yourself, or helping someone else)?

Please select all that apply:

- Yes – Phone call (e.g. a landline or your mobile phone)
- Yes – Video call (e.g. this includes Skype, Microsoft Teams, Facetime, WhatsApp, Zoom etc.)
- Yes – Email (e.g. discussing your care with a health professional)
- Other – please describe below.
- No

2. What were your experiences of the remote appointment using phone call/video call/email/other?

|  | Extremely bad | Quite bad | Neither good nor bad | Quite good | Extremely good |
| --- | --- | --- | --- | --- | --- |
| Usability of the technology (how easy or difficult was it to use) |  |  |  |  |  |
| Performance/Quality of the technology (audio, visual, connection etc.) |  |  |  |  |  |
| Usefulness/Helpfulness of the technology during appointment/consultation (did it help the conversation or allow you to understand things better) |  |  |  |  |  |

Please state anything else POSITIVE about the technology according to your experiences. Please state anything else NEGATIVE about the technology according to your experiences.

3. How do you feel about the use of technology (such as phones, or displays, or video calls, email) to support (not replace) consultations and appointments with health and care professionals?

|  | Not at all | Maybe for some things | Yes, for most things | Not decided yet |
| --- | --- | --- | --- | --- |
| Your willingness to use technology for consultations |  |  |  |  |
| Your ability to use the technology for consultations (you understand how to use it) |  |  |  |  |
| Access to/Availability of the technology for consultations (you have access to the equipment and connections needed) |  |  |  |  |

Please briefly tell us about any type of scenario/appointment/person where you would prefer to either: use technology where available or see the person face to face.

4. Have you ever heard of 3D (3 Dimensional) imaging or displays (seeing a 3D image or video of for example inside your body, your organs, or a scan)? Please select one that applies the most.

- I have never heard of this and would not be interested to use it
- I have never heard of this, but it sounds promising/interesting to use
- I have heard of it, but I am not interested in using it
- I have heard of this, and it sounds promising/interesting to use

Would you be willing to talk to a researcher and/or fill in any further surveys about 3D display technology for supporting remote appointments/consultations? If you would like to be entered into our prize draw for participants of this short questionnaire, please leave your email address below (there are 5 x £50 prize vouchers which will be selected at random from anyone who provides contact details). Please note, email addresses will only be used to notify winners of the draw and will be deleted – they are not attached or connected to your individual responses during analysis of our results.
