# Supplementary material for: Patient and Public Perceptions of 3D Technologies (Models and Images) to Facilitate Health Care Consultations: Exploratory, Mixed Methods Study
Source: JMIR Form Res. 2025 Jun 18;9:e65235. doi: 10.2196/65235 (PMC12192911; doi:10.2196/65235)
Supplement: Multimedia Appendix 3 [file formative-v9-e65235-s003.docx]

**Multimedia Appendix 3 - case study interview guide**

**INTRO**

**Who am I?**

**Who are the team?**

**What are we doing?**

**Did someone explain the purpose of this part of the study to you?**

**Do you understand that we will treat this information and confidential and de-identify you at all times when presenting our findings?**

**Do you have any questions before we proceed?**

**Are you happy to continue now with the interview?**

**START**

1. Tell me about your experience of 3D models (explain if they are not sure what you are referring to – do we need a print-out/screen shot as a reminder?) during your recent consultation.

- **Prompt:** Did you find the 3D model satisfactory, useful? Please explain.
- **Prompt:** Where were you, were you alone, were you able to handle the model, did you take it home?
- **Prompt:** How did it make you feel? Reduced/increased anxiety? Confident/less confident?
- **Prompt:** Do you think it had any impact on the relationship with your doctor? (for example, trust, confidence, communication, understanding)

1. What do you think the **benefits** are of using 3D models during a consultation?

- **Prompt:** understanding your illness, treatment options/risks involved, confidence (decision-making, sharing information with family/friends/beyond).

1. Can you think of any **barriers** of using 3D models during a consultation? If so, what comes to mind?

- **Prompt:** increased anxiety (too many details, information), confusing.

1. Do you feel 3D models should/could be implemented in future clinics or other healthcare settings during face-to-face consultations? Why/why not?

- **Prompt:** When would they be most useful? For example, at first diagnosis, in making treatment decisions, before/after surgery.

1. Do you have any additional comments/suggestions for the use of 3D models that you would like to share?

**<END>**

**OUTRO**

**What will happen next?**

**Do you have our contact details?**

**Any other questions?**

* At recruitment, participants will be encouraged to invite anyone they feel relevant to join them in the interview.
